# Employment Five Years After Cancer Diagnosis Among Native and Immigrant Women in Norway

**DOI:** 10.1007/s10926-025-10349-5

**Published:** 2025-12-06

**Authors:** Shahram Moradi, Steffen Torp, Roy Nielsen, Line M. Oldervoll, Harald K. Engan, Eliva Atieno Ambugo

**Affiliations:** 1 Research Group for Disability and Inclusion, Department of Health, Faculty of Health and Social Sciences, Social and Welfare Studies, University of South-Eastern Norway, Campus Porsgrunn, Porsgrunn, Norway; 2 Research Group for Health Promotion in Settings, Department of Health, Social and Welfare Studies, University of South-Eastern Norway, Borre, Norway; 3 Center for Crisis Psychology, Faculty of Psychology, University of Bergen, Bergen, Norway; 4 The National Institute on Intellectual Disability and Community, Department of Mental Health, Faculty of Medicine and Health Sciences, The Norwegian University of Science and Technology (NTNU), Trondheim, Norway; 5 Unicare Rehabilitation, Oslo, Norway; 6 The Fafo Institute for Labour and Social Research, Oslo, Norway

**Keywords:** Cancer survivorship, Employment disparities, Immigrant women, Employment participation, Immigrant employment

## Abstract

**Purpose:**

To compare employment status 5 years post-diagnosis among native Norwegian, Western immigrant, and non-Western immigrant female cancer survivors (CSs) and their matched controls without cancer, who were employed at the time of diagnosis.

**Methods:**

Participants were categorized into three groups based on data from Statistics Norway: Natives (CSs = 6587, control = 6587), Western immigrants (CSs = 209, control = 209), and non-Western immigrants (CSs = 105, control = 105). Women were aged 30–55 at diagnosis, employed (salaried/self-employed) at baseline, and alive at 5-year follow-up. CSs and controls were matched on age, education, and employment at diagnosis. Associations between cancer status, immigrant background, and employment were analyzed using binary logistic regressions and Firth penalized logistic regression to account for potential bias from small subgroup sizes.

**Results:**

At 5 years, female CSs had lower odds of employment compared with controls. Western immigrants did not differ from natives, whereas non-Western immigrants showed reduced employment. Higher income was positively associated with employment, while being married or cohabiting and working in the public sector were linked to higher odds. Interaction terms between cancer survivorship and immigrant background were not significant.

**Conclusion:**

Among women employed at baseline, cancer survivorship and non-Western immigrant background were independently associated with lower odds of employment five years later. Tailored employment support is needed for non-Western immigrants to improve long-term outcomes. Interventions should also target employers and workplaces to support employment maintenance among CSs.

## Introduction

Employment after cancer diagnosis is a challenge for many cancer survivors (CSs) [1]. The advent of precision medicine, targeted therapies, and immunotherapies have provided a new era in oncology that have resulted in increased longevity for individuals with cancer [2]. At present, cancer is perceived as a chronic condition rather than an immediate life-threating condition. This paradigm shift is associated with enhanced work ability and survivorship among people with cancer over the last decades [3, 4].

When people immigrate to a new country, they carry with them not only their cultural and economic backgrounds, but also their health profiles and challenges [5]. A report from the Norwegian Institute of Public Health [6] showed that immigrants bring diverse health profiles influenced by their countries of origin. The prevalence of certain health conditions varies significantly among different immigrant groups, underscoring the heterogeneity within the immigrant population. Studies have shown that immigrant women experience structural barriers in their employment, such as limited access to childcare, lack of recognition of foreign qualifications, job insecurity, wage disparities, and biases in employment [7–10]. For example, a qualitative study in Norway [11] found that Asian and African immigrant women on long-term sick leave due to chronic pain felt rejected in the workplace. They experienced exclusion and did not feel sufficiently needed or valued by their colleagues and employers when they attempted to re-enter employment after sick leave.

The proportion of immigrants in Norway has steadily increased from 4% in 1990 to 20% in 2023 [12]. Most labor immigrants in Norway came from European countries, followed by immigrants from Asian, African, and North American countries [13]. Fluency in Norwegian language, education level, and duration of stay in Norway are important factors for entry into the labor market for immigrants [14]. Immigrant women make up 16% of all resident women in Norway [15]. There is significant variation in education and work-related experiences among immigrant women, depending on their country of origin. However, immigrant women on average, possess lower education and work-related experiences compared to native Norwegian women [16]. Further, temporary employment for immigrant women is higher than for native women [17]. Kavli and Nicolaisen [16] also found that non-Western immigrant women experience less stable part-time employment in the Norwegian labor market compared to native women. However, they also have a higher transition rate to full-time positions, albeit with a slightly greater risk of exiting the workforce.

Immigrant women encounter distinct health challenges in accessing cancer screening services and clinical follow-up. These challenges are often linked to economic constraints, language barriers, insurance, trust, cultural differences with healthcare providers, or their specific migration status, such as being a refugee, labor immigrant, or family immigrant [18, 19]. A meta-analysis found that migrants are significantly less likely to be diagnosed with early stage cancer compared to non-migrants, suggesting lower engagement with preventive health services, including cancer counseling [20].

Butow et al. [21] conducted a meta-review of systematic literature reviews on return to work after a cancer diagnosis. They discovered that most studies have concentrated on women with breast cancer, highlighting a research gap concerning employment among women with other types of cancer. Addressing this research gap is particularly important for immigrant CSs, as they may face additional barriers beyond those experienced by the native population, including employment challenges, language difficulties, limited access to healthcare services, and socioeconomic constraints. Very few studies have previously investigated employment after cancer diagnosis among immigrant women, and the existing research has primarily concentrated on breast cancer [22, 23]. Blinder et al. [22] found that Latinas in the US were less likely to have returned to work 6 and 18 months following a breast cancer diagnosis compared to non-Latina whites. However, this employment disparity between the two groups was no longer evident after 36 months. In addition, Latinas experienced a longer duration before returning to work than non-Latina white women, indicating a slower employment recovery trajectory.

The aim of the present study is to compare employment status five years post-diagnosis among native women, Western immigrant women, and non-Western immigrant women CSs to matched controls without cancer, all of whom were employed at start of the study. In addition to these group comparisons, we examined whether differences remained after adjusting for key sociodemographic covariates, and whether the effect of cancer survivorship on employment varied by immigrant background.

## Methods

### Material

Our research is part of the wider Cancer and Work (CANWORK) research project, which is investigating factors related to CSs’ employment [24] with data collected from the Cancer Registry of Norway and Statistics Norway’s FD-Trygd database. This database covers the entire working-age population in Norway. CSs were identified from the Cancer Registry and cancer-free comparison group was randomly drawn from the remaining general population using national registries from Statistics Norway and matched on age, sex, education, and employment at baseline to ensure comparability at baseline. Controls were individuals without a cancer diagnosis at baseline or during follow-up; those who received a cancer diagnosis during the follow-up period were excluded from the analytic sample. A pre-analytic sample of 22,190 (11,095 CSs and 11,095 controls) for the current study was selected from the master dataset that comprised 58, 683 individuals (CSs = 19,561 individuals and controls = 39,122 controls).

The CS group comprised all cases of invasive cancer diagnoses (excluding basal cell carcinomas) defined by use of ICD-O-2 diagnostic codes between 2004 and 2008, aged between 30 and 55, and employed (either self-employed or salaried) at the time of diagnosis (baseline = T0). In addition, they were still living 5 years after the diagnosis (T5). We chose CSs and controls between the ages of 30 and 55 to be assured that the individuals had an established presence in the workforce. (i.e., neither younger and involved in education or associated activities, nor older and approaching retirement/early retirement due to illness/disability).

The following individuals were excluded from the master dataset: those lost to follow-up due, lack of registration, or out-migration (*n* = 289); individuals who passed away between T0 and T5 (*n* = 5031: 4661 CSs, 370 controls); duplicate records among controls (*n* = 290); cases with missing values on study measures (*n* = 4832); and individuals who were not employed at T0 (*n* = 7433). The remaining 40,808 participants (11,203 CSs, and 29,605 controls) comprised the basis for one-to-one Coarsened Exact Matching (CEM), which was performed on age, gender, education, and employment at T0. CEM, a statistical procedure designed to reduce imbalance between comparison groups in observational studies [25], was applied in a one-to-one ratio, providing the pre-analytic sample (*n* = 22,190: 11,095 CSs, 11,095 controls). Our study has been further limited to women only who in employment at the time of diagnosis (*n* = 13802: 6901 CSs, 6901 controls).

### Variables

The primary variables in this study included cancer survivorship, immigrant status (based on region of birth), and employment status at 5-year follow-up. In this study, we use the term employment status rather than return to work, as registry data only allow us to determine whether participants were employed at follow-up, and not whether they resumed their pre-diagnosis employment [26].

*Cancer survivorship* was a categorical variable with “no” (0 = cancer-free control) and “yes” (1 = CS).

*Immigrant status* (based on region of birth) was categorized into native (= 0), Western immigrant (= 1) and Non-Western immigrant (= 2). Those who were born in Norway from Norwegian parents or were born abroad with one or two parents born in Norway were categorized as natives, according to the definition by Statistics Norway. Western immigrants were those who immigrated to Norway from eastern and Western European countries, North America, and Oceania. This category includes countries such as Sweden, Germany, Poland, Russia, the United Kingdom, the United States, Canada, Australia, and New Zealand. Non-Western immigrants were those who immigrated from Africa, Asia, the Middle East, and South and Central America. Examples of these countries are Somalia, China, Iran, and Brazil. Thus, all the Western and non-Western immigrants in this study were first-generation immigrants to Norway.

### Statistical Analysis

Employment status at 5 years (T5) was the dependent variable, coded dichotomously (1 = employed, 0 = not employed). Immigrant status was analyzed as a categorical variable with three categories: natives, Western immigrants, and non-Western immigrants, with natives serving as the reference group. Two models were specified. Model 1 included cancer survivorship (CS vs. control) and immigrant status (natives, Western immigrants, non-Western immigrants). Model 2 extended Model 1 by adding sociodemographic covariates (income quartiles, marital status, children, and employment industry) and included interaction terms between cancer survivorship and immigrant status.

For descriptive purposes (Table 1), variables were presented in detail. Group differences between CSs and controls within each immigrant background group were examined using independent-samples t-tests for age (continuous variable) and chi-square tests for categorical variables at T0. These analyses were conducted to assess the presence of any baseline differences between groups, as presented in Table 1. For regression analyses (Table 2), some categories were combined to avoid very small cell sizes and to ensure more stable and interpretable estimates. For the employment industry variable, as shown in Table 2, all detailed industry categories presented in Table 1 were categorized into two main groups: public sector (including education, health, and public administration) and market/other sectors (including manufacturing, retail, transportation, and other industries).

**Table 1:** Sample characteristics (*n* = 13802)

	Natives		Western immigrants		Non-Western immigrants		Total	
	CSs	Control	CSs	Control	CSs	Control	CSs	Control
	*N* = 6587	*N* = 6587	*N* = 209	*N* = 209	*N* = 105	*N* = 105	*N* = 6901	*N* = 6901
Characteristic	%/mean	%/mean	%/mean	%/mean	%/mean	%/mean	%/mean	%/mean
Age_T0^a^ (years)^b^	46.30 (6.60)^c^	46.31 (6.60)^c^	46.21 (6.52)^c^	46.25 (6.47)^c^	42.98 (6.60)^c^	42.98 (6.60)^c^	46.24 (6.60)^c^	46.25 (6.61)^c^
Education_T0^d^								
Primary/unknown	17.7	17.7	11.0	11.0	33.3	33.3	17.7	17.7
Secondary	43.7	43.7	27.3	27.3	36.2	36.2	43.1	43.1
Tertiary	38.6	38.6	61.7	61.7	30.5	30.5	39.2	39.2
Marital status_T0								
Married/cohabiting	60.3	60.6	66.5	67.9	73.3	72.4	60.7	61.0
Separated/divorced	17.4	17.4	15.3	16.3	16.2	20.0	17.4	17.4
Widowed	1.5	1.4	1.4	1.0	1.9	1.9	1.6	1.3
Single	20.7	20.7	16.7	14.8	8.6	5.7	20.4	20.3
Children_T0								
No child	12.6	11.4	22.0	24.4	24.8	20.0	13.0	11.9
Child age 0–5	10.4	11.7	13.9	10.5	13.3	17.1	10.6	11.8
Child age 6–18	41.5	40.7	38.3	44.5	42.9	47.6	41.4	40.9
Child age 19 +	35.5	36.3	25.8	20.6	19.0	15.2	35.0	35.5
Income_T0								
1st quartile	19.6	20.8	18.7	25.8	35.2	25.7	19.8	21.0
2nd quartile	30.8	31.1	28.7	24.9	28.6	35.2	30.7	31.0
3rd quartile	29.9	29.1	29.7	23.0	24.8	25.7	29.8	28.9
4th quartile	19.8	19.0	23.0	26.3	11.4	13.3	19.8	19.1
Employment industry_T0								
Agriculture, forestry, fishing	1.0	1.0	1.0	0.5	0.0	1.0	1.0	0.9
Manufacturing	6.3	6.3	7.7	6.7	11.4	3.8	6.4	6.2
Construction	1.1	1.3	1.0	0.0	0.0	1.0	1.1	1.2
Wholesale/retail/auto repair	10.7	11.5	9.1	6.2	4.8	11.4	10.5	11.3
Transport, Storage	3.9	3.7	1.9	3.3	1.0	1.9	3.8	3.7
Accommodation, food service	1.5	1.9	3.8	1.0	10.5	13.3	1.7	2.0
Admin, support services	6.8	6.8	9.6	9.1	10.5	3.8	6.9	6.8
Public admin, defence	8.0	8.3	5.7	3.3	6.7	8.6	7.9	8.2
Education	12.9	12.8	15.8	15.8	9.5	7.6	13.0	12.8
Human health, social work	35.6	35.3	36.4	39.7	39.0	41.9	35.7	35.5
Other industries	8.7	8.7	6.2	10.0	1.9	1.9	8.5	8.7
Unknown industry	3.4	2.6	1.9	4.3	4.8	3.8	3.3	2.6
Length of stay in Norway								
2 years or less	–	–	1.9	2.9	1.9	1.9	–	–
3–5 years	–	–	7.2	7.7	9.5	8.6	–	–
6–10 years	–	–	16.3	20.6	16.2	19.0	–	–
11–20 years	–	–	42.1	34.4	43.8	46.7	–	–
21 years and more	–	–	32.5	34.4	28.6	23.8	–	–
Cancer malignancy_T0								
Local	53.2	–	47.4	–	42.9	–	52.8	–
Regional	22.6	–	27.3	–	23.8	–	22.7	–
Distant	2.6	–	2.9	–	2.9	–	2.6	–
Unknown	21.7	–	22.5	–	30.5	–	21.9	–
Cancer type/diagnosis_T0								
Buccal cavity & pharynx^e^	0.2	–	0.5	–	1.0	–	0.2	–
Digestive organs	6.2	–	5.7	–	2.9	–	6.1	–
Respiratory, intrathoracic organs	1.0	–	1.9	–	0.0	–	1.0	–
Bone, articular cartilage	0.1	–	0.0	–	0.0	–	0.1	–
Blood^f^	2.4	–	1.9	–	6.7	–	2.4	–
Skin^g^	12.9	–	5.3	–	1.9	–	12.5	–
Mesothelial, soft tissue	0.7	–	0.0	–	0.0	–	0.7	–
Breast	41.8	–	39.7	–	38.1	–	41.7	–
Female genital organs	14.1	–	19.1	–	18.1	–	14.3	–
Urinary tract	1.8	–	2.4	–	1.0	–	1.8	–
Eye, brain & other CNS^h^	8.2	–	10.5	–	6.7	–	8.2	–
Thyroid, other endocrine glands	5.4	–	6.2	–	17.1	–	5.6	–
Ill-defined, secondary, unspecified sites	3.1	–	2.4	–	3.8	–	3.1	–
Working_T5^i^ (/no)	86.3	91.5	84.7	89.5	73.3	84.8	86.0	91.3

Values are presented as means (SD) or percentages. Group differences between CSs and controls at T0 showed no statistically significant differences across groups, except for the variable Children in the Norwegian group, which showed a significant difference (*χ*^2^ = 9.59, *p* = 0.022). ^a^At diagnosis; ^b^Range: 30–55years old; ^c^Standard deviation, ^d^Education levels are categorized as follows: Primary education includes elementary schooling or no recorded education. Secondary education refers to high school or equivalent. Tertiary education includes higher education, such as university or college. ^e^Lip, oral cavity, pharynx, ^f^Haematopoietic & reticuloendothelial tissues, ^g^Including malignant melanoma but not basal cell carcinomas; ^h^other CNS = other parts of the central nervous system; ^i^5 years post-diagnosis; *CSs* cancer survivors

**Table 2:** Logistic and Firth regression models predicting employment five years after cancer diagnosis: n = 13802

Predictor	Model 1 Logistic OR (95% CI)	Model 1 Firth OR (95% CI)	Model 2 Logistic OR (95% CI)	Model 2 Firth OR (95% CI)
Cancer status				
CSs vs. controls	0.59 (0.53–0.65)***	0.59 (0.53–0.65)***	0.57 (0.51–0.63)***	0.57 (0.51–0.63)***
Immigrant background at T0				
Natives	1.00 (ref)	1.00 (ref)	1.00 (ref)	1.00 (ref)
Western immigrants	0.84 (0.63–1.13)	0.84 (0.63–1.13)	0.75 (0.49–1.22)	0.74 (0.48–1.19)
Non-Western immigrants	0.47 (0.33–0.66)***	0.47 (0.34–0.66)***	0.54 (0.32–0.97)*	0.53 (0.31–0.94)*
Cancer status × Western immigrant	–	–	1.09 (0.59–1.97)	1.09 (0.60–1.97)
Cancer status × Non-Western immigrant	–	–	0.87 (0.42–1.75)	0.88 (0.43–1.76)
Income at T0				
1st quartile			1.00 (ref)	1.00 (ref)
2nd quartile	–	–	1.80 (1.57–2.07)***	1.80 (1.57–2.07)***
3rd quartile	–	–	2.54 (2.19–2.96)***	2.54 (2.18–2.95)***
4th quartile	–	–	3.18 (2.72–3.73)***	3.17 (2.71–3.72)***
Marital Status at T0				
Married/cohab (vs other)	–	–	0.70 (0.62–0.79)***	0.70 (0.62–0.79)***
Having children at T0				
Children (yes vs none)	–	–	0.99 (0.84–1.17)	0.99 (0.84–1.17)
Employment industry at T0				
Public sector (vs private/other)	–	–	0.58 (0.52–0.64)***	0.58 (0.52–0.64)***
Constant	10.75***	10.74***	9.16***	9.13***
Nagelkerke R^2^ (for logistic regression)	0.016	–	0.069	
LR *χ*^2^ (for Firth regression)	–	115.14	–	493.14

*OR* odds ratio; *CI* confidence interval; *CSs* cancer survivors

^***^p < .001, **p < .01, *p < .05

Associations were first analyzed using standard binary logistic regression. To assess robustness and address potential small-sample bias in immigrant subgroups, we also conducted Firth penalized logistic regression. Odds ratios (ORs) with 95% confidence intervals (CIs) were reported for both approaches. Model fit was assessed using Nagelkerke *R*^2^ for binary logistic regression and likelihood ratio *χ*^2^ tests for Firth regression.

Because age and education were used as matching variables at baseline, they were not included as covariates in the regression analyses to prevent redundancy and overadjustment. However, descriptive information on age and education is presented in Table 1 to illustrate baseline comparability between groups. Other potential covariates (e.g., cancer type, metastasis status, and length of stay in Norway) were excluded due to limited subgroup sizes, especially among non-Western immigrants. Including these variables would have reduced statistical power, increased risk of overfitting, and in some cases prevented model convergence. All analyses were conducted in R (version 4.5.1) and SPSS (version 31). Statistical significance was set at *p* < 0.05.

We also explored whether employment trajectories could be identified using sequence analysis or related longitudinal clustering approaches. However, the available data included only six annual binary employment observations (T0–T5), which limited the ability to form interpretable trajectories. Initial attempts at sequence-based clustering and mixed-effects models produced non-convergent estimates, particularly for small immigrant subgroups. Therefore, we focused on binary and Firth logistic regression models for the purpose of this study.

### Ethics

The present research was approved by the Norwegian Data Inspectorate (number: 16/00235), the Norwegian Social Science Data Services, and the Regional Committee for Medical Research Ethics of Mid-Norway (number: 2016/830). The current study, which is based on secondary data from Norwegian registries, did not require written consent from participants. Researchers who intend to use these data must obtain approval from the above registries and agencies.

## Results

Table 1 presents the basic characteristics of natives, Western and non-Western female immigrant CSs and the matched control groups. Baseline characteristics were well balanced between CSs and controls across all variables, with no significant differences observed except for the “children” variable among Norwegian participants (*χ*^2^ = 9.59, *p* = 0.022). The mean age at diagnosis was 46 years for natives and Western immigrants, whereas non-Western immigrants were slightly younger, with a mean age of around 43 years. Education levels varied across groups, with Western immigrants having the highest proportion of tertiary education (61.7%), followed by natives (38.6%) and non-Western immigrants (30.5%). Non-Western immigrants had the highest proportion with primary or unknown education (33.3%).

Marital status differences were observed, with non-Western immigrants more likely to be married or cohabiting (73.3%) than Western immigrants (66.5%) and natives (60.3%). Income disparities were evident, as 35.2% of non-Western immigrant CSs fell into the lowest income quartile, compared to 19.6% of natives and 18.7% of Western immigrants. Employment sectors also varied, with non-Western immigrants overrepresented in industries such as accommodation and food services (10.5%) and administrative support (10.5%), while natives and Western immigrants had higher representation in education and public administration.

Regarding cancer characteristics, breast cancer was the most common diagnosis across all groups (around 40%), but non-Western immigrants had a higher prevalence of blood cancers (6.7%) and thyroid cancer (17.1%) compared to the other groups. In addition, non-Western immigrants had the highest proportion of cases with unknown cancer malignancy (30.5%). Finally, employment five years post-diagnosis were lowest among non-Western immigrant CSs with 73.3%, compared to 84.7% of Western immigrants and 86.3% of natives.

**Fig. 1:**
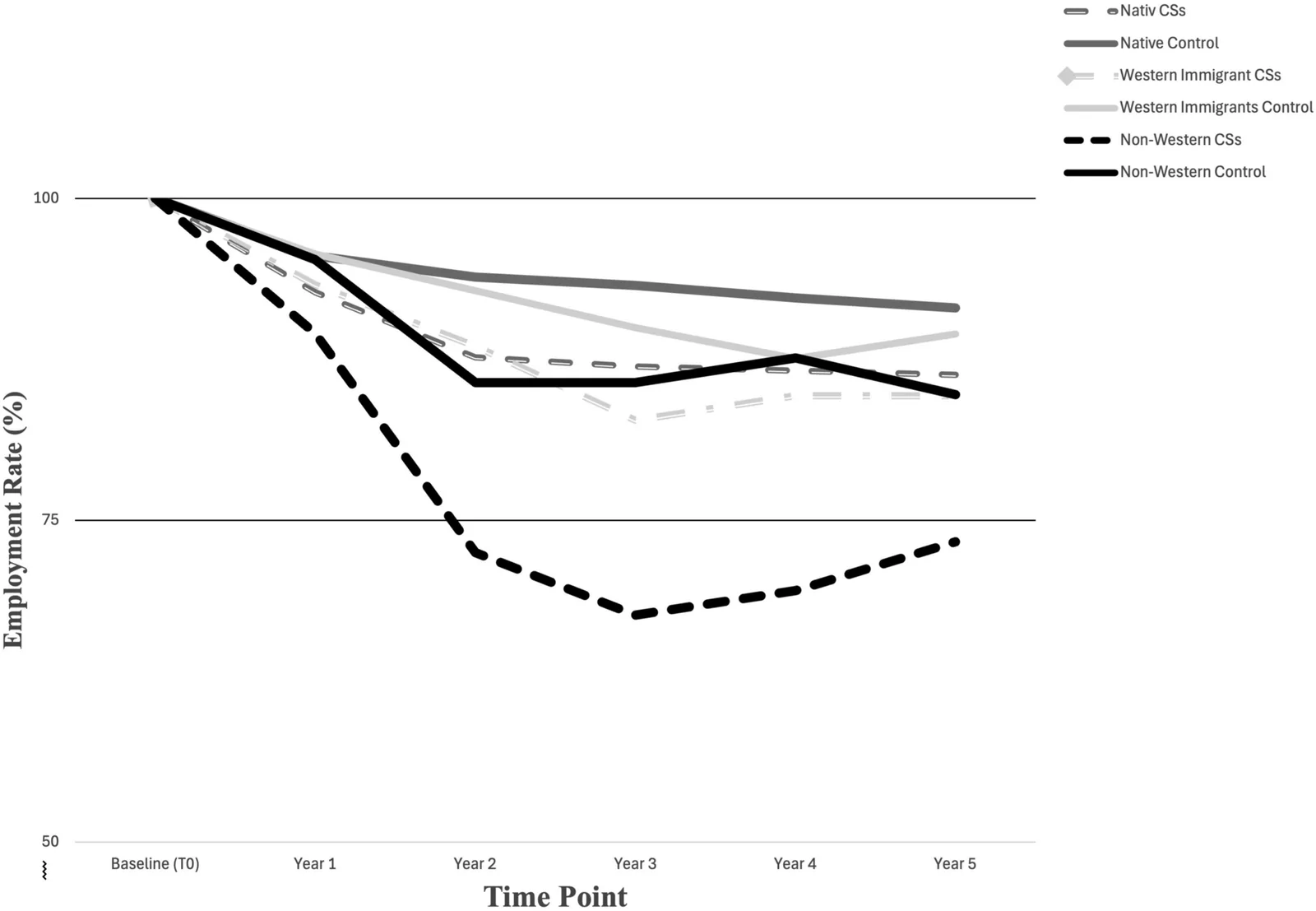
Employment trends of both cancer CSs and control groups across different immigrant backgrounds over a 5-year period

Figure 1 shows the percentage of CSs and controls employed rates over a 5-year period, separated by immigrant background (Natives, Western immigrants, and non-Western immigrants). Across the 5-year follow-up period, a decline in employment rates was observed for both CSs and controls. The extent of this decline varied by group (CSs and controls) and immigrant background, highlighting potential disparities in employment retention.

Among controls, natives and Western immigrants maintained relatively high employment rates over time, with employment remaining above 85% at year five. In contrast, non-Western immigrant controls exhibited a sharp decline until two years after diagnosis, after which employment levels stabilized.

Among CSs, non-Western immigrants experienced the sharpest decline in employment rates, particularly during the first three years after cancer diagnosis. By 3 years after diagnosis, non-Western CSs had the lowest employment compared to natives and Western immigrant CSs. However, after three years, employment rates among non-Western immigrant CSs showed signs of stabilization or slight recovery, whereas employment among the other CS groups remained relatively stable.

Table 2 presents the results from logistic and Firth penalized regression. In both approaches, CSs had significantly lower odds of employment five years after baseline compared with controls (Model 1: OR = 0.59, 95% CI [0.53, 0.65]; Model 2: OR = 0.57, 95% CI [0.51, 0.63]).

Regarding immigrant status, Western immigrants did not differ significantly from natives, while non-Western immigrants consistently showed reduced odds of employment. In the Firth regression models, non-Western immigrants had significantly lower odds of being employed compared to natives (Model 1: OR = 0.47, 95% CI [0.34, 0.66]; Model 2: OR = 0.53, 95% CI [0.31, 0.94]).

According to the Firth regression results (Model 2), higher income at baseline was strongly associated with greater odds of employment, showing a clear gradient across quartiles (e.g., OR = 3.17, 95% CI [2.71, 3.72] for the highest vs. lowest quartile). Being not married or cohabiting was linked to lower odds of employment (OR = 0.70, 95% CI [0.62, 0.79]), and working in private/ other sectors was associated with lower odds compared with the public sector (OR = 0.58, 95% CI [0.52, 0.64]). Having children was not significantly associated with employment.

The interaction terms between cancer survivorship and immigrant status included in Model 2 were not statistically significant, indicating that the effect of cancer on employment did not differ by immigrant background.

Model fit improved when socioeconomic covariates were included: for logistic regression, Nagelkerke R^2^ increased from 0.016 (Model 1) to 0.069 (Model 2); for Firth regression, the likelihood ratio *χ*^2^ increased from 115.14 (Model 1) to 493.14 (Model 2).

## Discussion

This study examined the association between cancer survivorship, immigrant background, and employment status 5 years post-diagnosis among women in Norway. Our descriptive findings (Fig. 1) highlight disparities in employment trajectories among CSs during the first 5 years post-diagnosis. Of note, employment percentages at each time point (from diagnosis to 5 years after) are cross-sectional, indicating that some individuals may have moved in and out of employment during the period. Non-Western immigrant CSs experienced the steepest decline in employment during the first 3 years, followed by a gradual improvement thereafter. We argue that this pattern reflects the importance of the early post-diagnosis period, as employment loss is most pronounced during this time. We reason that the subsequent recovery may be attributed to factors such as treatment completion, adaptation to work demands, or increased support from employers and colleagues at work, even though this group continued to have the lowest overall employment rates following cancer diagnosis. These interpretations highlight the need for further research to explore the underlying mechanisms driving these employment patterns.

The regression analysis revealed that both cancer survivorship and non-Western immigrant background were independently associated with decreased odds of employment. Specifically, CSs had 43% lower odds of being employed compared to the control group. Western immigrants did not differ significantly from natives, whereas non-Western immigrants had substantially lower odds of employment. Notably, there was no significant interaction between cancer survivorship and immigrant background, suggesting that the impact of cancer survivorship on employment is consistent across different immigrant backgrounds. Our findings are in line with previous research indicating that, despite advancements in cancer treatment, long-term employment retention continues to be a significant concern for female CSs [3, 27].

Thøgersen et al. [28] found that non-Western immigrants in Norway experience longer waiting times for breast and lung cancer treatment, which may delay recovery and negatively affect employment outcomes. Additionally, Bhargava et al. [29] reported that non-Western immigrant women had a higher proportion of aggressive tumor profiles, such as larger tumor sizes, higher histologic grade, and increased lymph node involvement compared to non-immigrants. We reason that these disparities in healthcare access and tumor severity may be associated with delayed diagnosis, more intensive treatment, and prolonged recovery periods. Although we did not find a significant interaction between cancer survivorship and immigrant background in employment outcomes, future research should investigate whether enhancing early detection could improve long-term employment outcomes for immigrant CSs.

Another explanation for the lower employment rates among non-Western immigrant women, which applies to both CSs and controls, is their overrepresentation in lower-paying jobs with limited workplace support [30, 31]. Although nearly all employees in Norway qualify for sickness benefits after four weeks of employment, immigrant women are disproportionately represented in jobs offering limited flexibility and few workplace accommodations [16]. Nevertheless, Norway’s universal welfare system, including comprehensive medical care and sickness benefits, likely provided important protections for many immigrant women in this study.

Additionally, systemic barriers such as limited language proficiency, cultural differences, and potential discrimination in the labor market may further impede immigrants` employment prospects following a cancer diagnosis [14, 30, 32]. Another disadvantage for non-Western immigrant women is their lower awareness of psychological support services; for instance, Singer et al. [33] found that CSs from the near and Middle East in Germany were significantly less aware of psychotherapy services compared to native-born individuals. This lack of awareness can impede their access to crucial psychosocial support during and after cancer treatment, which may negatively affect their overall well-being and ability to sustain employment.

During the time period when our data were collected (2004–2008), it is likely that a substantial proportion of non-Western immigrants in Norway included refugees who had fled conflict or persecution. Although exact figures for this period are unavailable, previous reports indicate that Norway has consistently received a significant number of refugees [34]. Given the diversity within non-Western immigrant groups, it is important for future research to study how their varied experiences, particularly those related to refugee status, influence their employment trajectories.

We hypothesize that the lack of significant difference in employment outcomes between Western immigrant women and native women may be attributed to the reasons for immigration and the extent of cultural and linguistic integration. Many Western immigrants come to Norway as labor migrants, which may facilitate their integration into the workforce. This is supported by studies showing that immigrants from Western European countries tend to achieve better labor market outcomes than those from Eastern Europe or non-Western countries [35]. Leschke and Weiss [35] found that mobile workers from EU-West countries obtain better jobs and wages than those from EU-East countries, highlighting the existence of labor market hierarchies among intra-EU migrants.

In our study, Western immigrants include individuals from both Western and eastern European countries, North America, and Oceania. While there may be cultural and linguistic differences compared to natives, particularly with immigrants from Eastern Europe, these individuals often possess skills and qualifications that are in demand in Norway. Moreover, the initial jobs that immigrants obtain can significantly influence their long-term labor market integration [36]. Western immigrants (as defined in the current study) may have better access than non-western immigrants to positions that match their skills and qualifications, facilitating smooth integration and better employment outcomes over time [36]. Their educational qualifications and professional experiences are likely to be recognized and valued in Norway, which can mitigate employment barriers.

Our findings regarding Western immigrant women necessitate further investigation. While our quantitative data suggests similar employment outcomes between Western immigrants and natives, it fails to capture the unique challenges of these women. We speculate that Western immigrant women often bring work-related experiences shaped by their country of origin, including professional qualifications and skills that align with labor market demands in Norway. What makes their experiences unique is the relative ease of transferring qualifications and cultural alignment with Norwegian workplace norms, compared to non-Western immigrant women. However, they may still face challenges such as navigating a new labor market, language barriers, or adapting to differences in workplace dynamics and expectations. Future qualitative research could offer deeper insights into the labor market integration of Western immigrant women in Norway, examining potential differences between CSs and those without a cancer diagnosis. Such studies might explore individual experiences, workplace dynamics, and the role of social support networks in facilitating continued labor market participation.

Socioeconomic and occupational factors also played an important role: higher income increased the likelihood of continued employment, whereas being married or cohabiting and employment in the public sector were associated with higher odds. These findings indicate that financial resources, family responsibilities, and workplace context independently impact employment among women in this study. However, the ways in which these factors interact with cancer survivorship and immigrant background are likely to be more complex than our analyses could capture. Addressing such interactions would require larger samples, particularly among immigrant populations, or longitudinal designs that follow individuals over time.

### Implications for Clinical Practice and Migration Policy

The current study underscores the necessity for tailored employment support programs, particularly for non-Western immigrant women, given the structural labor market barriers affecting both CSs and non-CSs. Healthcare providers and the Norwegian Labour and Welfare Administration (NAV) should play a central role by offering language-specific resources and integrating vocational rehabilitation services into survivorship care plans. Health professionals, including occupational therapists and rehabilitation specialists, can play a key role in supporting patients’ labor market participation by identifying work-related challenges early, coordinating with employers, and facilitating tailored rehabilitation plans. Employers likewise play a crucial role by providing flexible work arrangements and accommodations that address the specific needs of CSs in maintaining employment. Such accommodations may include gradual return-to-duty schedules and adapted tasks.

From a broader perspective, these findings also have implications for migration policy. In Norway, labor market participation is a central dimension of integration efforts. Our results indicate that cancer may constitute an additional vulnerability factor for non-Western immigrant women, potentially reinforcing existing inequalities in employment. Addressing this challenge requires coordinated action not only between healthcare services and NAV, but also across other key institutions such as labor market authorities, the Directorate of Integration and Diversity (IMDi), and the Norwegian Directorate of Immigration (UDI). Strengthening cross-sector collaboration and ensuring that rehabilitation and integration policies are mutually reinforcing may help maintain employment among non-Western immigrant female CSs and promote their long-term social and economic inclusion.

### Strengths and Limitations of the Study

This study is among the pioneering efforts to examine long-term employment outcomes among female CSs with both Western and non-Western immigrant backgrounds in Norway and the Western world, thereby addressing a significant gap in the existing research literature. Secondly, the use of large-scale, good quality national registry data enhances the statistical power and generalizability of the findings while minimizing selection biases.

Several limitations should be considered when interpreting the findings. CEM was selected for its ability to achieve strong balance with minimal model dependence while retaining more observations than traditional exact matching [37]. However, other alternative methods such as propensity score matching (PSM) or inverse probability weighting (IPW) could also have been applied for matching CSs and controls. We recommend that future studies consider comparing results across different matching strategies to assess the robustness of results and explore whether adding additional continuous variables (e.g., income based on annual salary) affects employment outcomes.

This study specifically focused on individuals who were employed at baseline, as the aim was to assess long-term employment outcomes among those actively participating in the labor market at the time of cancer diagnosis. While this approach enables an analysis of being employed five years after diagnosis, it excludes individuals who were unemployed at baseline, potentially limiting the generalizability of the findings to the broader population of CSs. Additionally, the study includes only CSs who were alive five years post-diagnosis. Including individuals who passed away during the follow-up period might yield different findings, as these individuals could have experienced more severe disease progression or other factors influencing employment outcomes. Information on working hours (e.g., full-time vs. part-time) at baseline was not available. Future studies could include this measure, as full-time employment may reflect stronger labor market attachment, whereas part-time work may represent either a health-related adaptation or a step toward full participation. Future research should also examine employment trajectories using larger and more diverse samples of CSs, including those not employed at baseline or who do not survive long-term. More detailed analyses of immigrant populations are also needed, as the broad categories of Western and non-Western immigrants may mask important differences related to country of origin, length of stay in Norway, language proficiency, or access to social and occupational support.

## Conclusion

This study demonstrates that, among women who were employed at the time of cancer diagnosis, both cancer survivorship and a non-Western immigrant background were independently associated with lower employment rates 5 years later, whereas Western immigrant women showed outcomes comparable to native women. These disparities persisted after adjusting for socioeconomic and occupational factors, suggesting that structural labor market barriers and/or unequal access to health services may play an important role. The findings underscore the need for targeted employment and rehabilitation interventions that address the intersection of health and migration.

## Data Availability

The data used in this study are derived from national administrative registries (e.g., Statistics Norway, the Norwegian Cancer Registry). These data are not publicly available due to privacy and data protection regulations, but may be made available to qualified researchers upon reasonable request and subject to approval by relevant ethical and legal authorities.
